# Different dimerisation mode for TLR4 upon endosomal acidification?

**DOI:** 10.1016/j.tibs.2011.11.003

**Published:** 2012-03

**Authors:** Monique Gangloff

**Affiliations:** Department of Biochemistry, University of Cambridge, 80 Tennis Court Road, Cambridge CB2 1GA, UK

**Keywords:** Toll-like receptors, TLR4, MD-2, LPS, MPLA, Mal/TIRAP, MyD88, TRAM, TRIF, lipid raft, conformational change, dimerisation, signalling, endosomal acidification

## Abstract

TLR4 is unique among pathogen-recognition receptors in that it initiates different pathways in different cellular locations. Binding of a bridging factor, Mal, allows recruitment of an adapter protein, MyD88, at the plasma membrane, which leads to the production of proinflammatory cytokines. Upon internalization, TLR4 uses a different bridging factor, TRAM, to activate a MyD88-independent pathway that results in type I interferon expression. Interestingly, both Mal and TRAM are localised initially at the plasma membrane. In this Opinion, I suggest a possible mechanism by which endosomal acidification triggers the differential adaptor usage of TLR4. I discuss the evidence of the pH sensitivity of TLR4 and propose a new dimerisation mode for TLR4 based on the crystal structure of the related receptor TLR3 bound to its ligand, double-stranded RNA.

## TLR signalling

**Figure 1:**
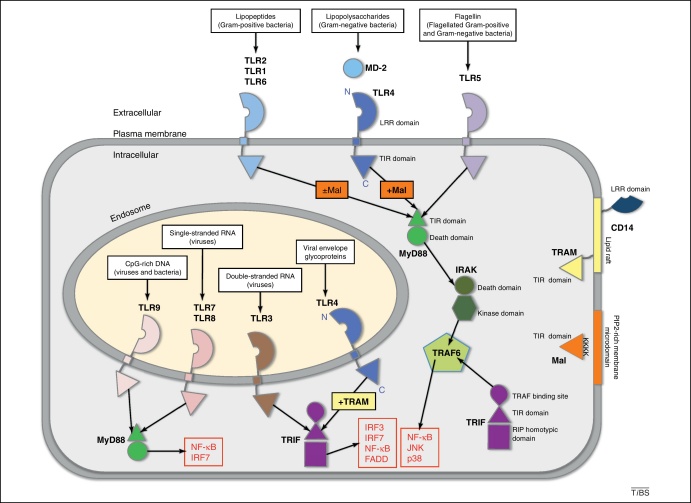
TLR signalling. There are two types of TLRs, those located at the plasma membrane that sense microbial membrane components and the intracellular ones that sense microbial nucleic acids. TLR4 can signal both at the plasma membrane and at endosomes, where it can be activated by viral envelope glycoproteins. All TLRs signal via the adaptor MyD88, except TLR3 that can function only via TRIF. MyD88 leads to the activation of kinases such as IL-1 receptor-associated kinases (IRAKs). The resulting phosphorylation cascade activates members of TNF receptor associated factor 6 (TRAF6) and, ultimately, transcription factors NF-κB, JNK and p38 (as well as IRF7 for TLR7 and TLR9). The TRIF pathway stimulates TRAF6, Fas-associated protein with death domain (FADD) and the transcription factors IRF3, IRF7, NF-κB. TLR4 is the only receptor that can engage both MyD88-dependent and -independent pathways. It requires bridging adaptors TRAM and Mal to recruit MyD88 and TRIF, respectively. TLR2 depends only partially on Mal. Membrane signalling triggers an inflammatory response whereas intracellular TLR signalling leads to antiviral and adjuvant responses.

Toll-like receptors (TLRs) are key regulators of the innate immune response to pathogens. They recognize signature molecules that are crucial for the integrity of the microorganism and distinguishable from self either by their chemical composition or by their cellular localisation (Fig. 1) [1]. For example, viral and bacterial nucleic acids are picked upon in endolysosomal compartments but not in the cytoplasm or the extracellular space of the host. This is achieved by the nucleic acid-binding subgroup of TLRs (TLR3, TLR7, TLR8 and TLR9) that are expressed only intracellularly. In contrast, microbial cell wall components such as lipopolysaccharides (LPS), lipopeptides and flagellar proteins are recognized at the plasma membrane of immune and epithelial cells by TLR4, TLR2 and TLR5, respectively. Ligand binding to TLRs is the first event in a signalling process that allows defences to be put in place for clearance of the infection and for long-term protection via the molecular memory of the adaptive immunity.

TLRs are type I membrane receptors (see Glossary) with extracytoplasmic domains (ectodomains) responsible for ligand binding. These domains contain versatile leucine-rich repeats (LRRs) that mediate binding and form a right-handed superhelix that extends throughout the domain and adopts the shape of a horseshoe. TLR4 is the only TLR that requires a co-receptor to bind a ligand; the co-receptor is a small lipid-binding glycoprotein known as myeloid differentiation factor 2 (MD-2). In addition to LPS, TLR4 can bind other ligands, such as various small molecules as well as endogenous or exogenous proteins, in the presence or absence of MD-2 [2].

Signalling downstream of TLRs is initiated by either homodimerisation or heterodimerisation of the receptor; for example, TLR3 and TLR4 form homodimers, whereas TLR2 forms heterodimers with TLR1 or TLR6 [3]. Ligand binding induces a crosslinking of the ectodomains, which in turn causes the intracellular signalling domains of the receptors to be brought in close proximity. The signalling domain comprises the Toll-IL-1 receptor (TIR) domain, which is present also in the intracellular adaptor proteins MyD88 and TRIF, and their bridging factors Mal (also known as TIRAP) and TRAM [4]. These adapters and bridging factors are platforms that organize downstream signalling. TIR domains do not possess enzymatic activity but function via protein-protein interactions to recruit cellular kinases that activate specific transcription factors. As individual TIR domains do not have detectable affinity for each other, it is likely that the assembly of the signalling complex triggers the juxtaposition of multiple TIR domains in order to increase affinity by a crowding effect [5].

MyD88 is attracted to the activated receptors via its C-terminal TIR domain. This adaptor is used by all TLRs, except TLR3. Engagement of MyD88 initiates polymerisation of its N-terminal death domain (DD) into a left-handed helical structure made of six to eight subunits of MyD88, 4 subunits of the IL-1-receptor-associated kinase (IRAK)4, and four molecules of either IRAK1 or IRAK2. This supramolecular assembly is referred to as the myddosome [6,7]. In contrast to the TIR interactions, the assembly of DDs forms a stable complex. The DD signalling tower, in turn, leads to auto-phosphorylation or cross-phosphorylation of IRAKs, activation of TNF-receptor-associated factor (TRAF)6 and, subsequently, activation of nuclear factor (NF)-κB and mitogen-activated protein kinases (MAPKs). For TLR7 and TLR9, IRAK1 can also stimulate interferon-regulatory factor IRF7 to produce IFNα. By contrast, TLR3 exclusively recruits the adaptor TRIF (also known as TICAM1) via its TIR domain. TRIF possesses different protein interaction motifs that allow recruitment of TRAF6 and the protein kinases TBK1 and RIPK1, which in turn leads to the activation of another adaptor, Fas-associated protein with death domain (FADD), as well as the transcription factors NF-κB, IRF3 and IRF7, respectively.

Once more, TLR4 stands out compared to other members of the TLR family in that it initiates both MyD88-dependent and MyD88-independent pathways. This is achieved by using the bridging factors Mal and TRAM [8–12], respectively, at different times and locations (Figure 1) [13]. Initial binding of Mal allows TLR4 recruitment of MyD88 at the plasma membrane and signalling within a few minutes [14]. About half an hour later, TLR4 is translocated away from the plasma membrane, where TRAM is believed to help recruit TRIF at endolysosomes [13,15]. Both Mal and TRAM are subjected to tight regulatory controls by phosphorylation [16]. This provides extra control on the TLR4 signalling pathway and gives cellular context to the response elicited by a given ligand. Interestingly, both adaptors are localised at the plasma membrane via a phosphatidyl-inositol-4,5-bisphosphate (PIP2) binding motif for Mal [14] and a myristoyl anchor for TRAM, respectively [17]. Only TRAM shuffles between the cell membrane and endosomal vesicles, where it induces signalling. In this Opinion, I propose that TLR4 adopts different conformations in the two cellular locations, and that this feature allows the receptor to activate distinct pathways in each location.

## An alternative dimerisation mode for TLR4

**Figure 2:**
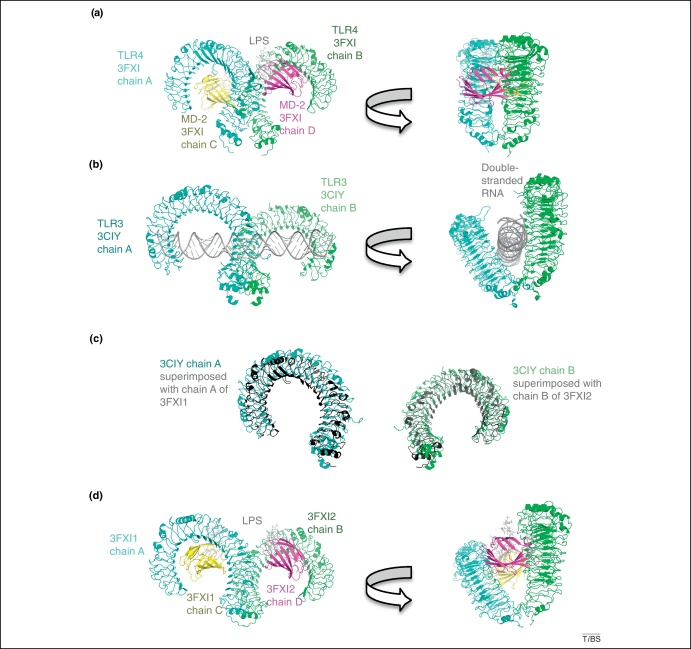
TLR ectodomains form m-shaped dimers that are either vertical or tilted. **(a)** The crystal structure of the complex formed by TLR4, MD-2 and LPS at pH 7.5 (PDB code 3FXI) forms a vertical dimer. **(b)** When bound to their ligand (double-stranded RNA) at acidic pH, TLR3 dimers are tilted (PDB code 3CIY). **(c)** Superimposed TLR4 and TLR3 ectodomains occupy similar spaces despite their different length and degree of curvature. TLR4 is shown in black and TLR3 in cyan and green. **(d)** A TLR4-MD-2-LPS dimer arranged in a TLR3-like tilted conformation was built in PyMol by superimposing receptor chain A of a first copy of 3FXI (3FXI1) onto chain A of 3CIY. Another superposition was achieved by using chain B of a second copy of 3FXI (3FXI2) onto chain B of the previous 3CIY. The primary interface between TLR4 and MD-2 was left intact. Supplementary Table S1 and Supplementary Figure S2 show that the primary binding site between TLR4 and MD-2 is pH-independent. The new dimer was produced by chains A and C from 3FXI1 and chains B and D from 3FXI2 (coordinates are available as Supplementary Material).

The initial observation that led to this article was the comparison of the m-shaped crystal structures of the ectodomains of the TLR3 dimer, the TLR2-TLR1 or TLR2-TLR6 heterodimers, and the TLR4 dimer, when bound to their respective ligands. Whereas the TLR4 homodimer and the TLR2 heterodimers adopt a vertical structure, the TLR3 homodimer is tilted (Figure 2a,b). Could this variation in tilt angle contribute to the observed functional differences in adaptor recruitment? Remarkably, the TLR4-MD-2-LPS complex was prepared at pH 7.5 [18], whereas the TLR3 complex was purified and crystallized at pH 5.5 (Supplementary Table S1) [19]. Because a crystal structure is a snapshot of a given molecular state in a well-defined environment, could the acidification of the TLR4 complex trigger the adoption of a tilted conformation similar to that reported for TLR3?

To try to answer this question, I used the molecular visualisation software Pymol [20] to generate *in silico* an arrangement of the TLR4-MD-2 complex that was similar to that of the TLR3 complex (Figure 2c,d). The coordinates of this hypothesized TLR4-MD-2-LPS model are available as a PDB file in the Supplementary Material. Despite the TLR4 ectodomain being about 70 residues and two LRRs shorter than that of TLR3, both molecules occupied similar spaces because of their different degree of curvature (Figure 2c). In this *in silico*-generated complex, the TLR4 dimer presents the same tilt-angle as TLR3, and MD-2 cross-links TLR4 chains in a manner that is less compact than the crystal structure and reminiscent of a model proposed by Kim *et al.* (Supplementary Figure S1) [21]. Interestingly, the N-linked glycosylations in TLR3 and TLR4, as well as LPS in the case of TLR4, were required for the superposition to generate a model without major atomic clashes. The structure of the TLR3 dimer and that of the *in silico*-generated TLR4 superimpose with an rmsd of 5.40 Å over 6440 atoms.

The alternative dimerisation site on TLR4 is located at the juxtamembrane region and involves the C-terminal LRR21 and the cysteine-rich capping region LRRCT. Importantly, the tilted ectodomains bring their C-termini closer together, with the α carbons of Cys627 about 6 Å apart instead of 22 Å. The size of the dimerisation surface in the *in silico*-generated model (580 Å^2^) is similar to that of the original TLR4 structure (610 Å^2^; PDB code 3FXI) However, the participation of MD-2 in the dimerisation surface is weaker in the TLR3-derived model (77 Å^2^ instead of 470 Å^2^), and the interacting residues are different. Whereas MD-2 interacts with LRR15 to LRR17 in the original TLR4 structure, it seems to interact with LRR17 and LRR18 in the TLR3-derived model. Interestingly, the common interface (LRR17) is centred on MD-2 residues Thr84 and Met85, in a region known to undergo conformational changes upon ligand binding (Supplementary Figure S2) and to confer constitutive activity in a species-specific domain-swapping study [22]. In addition, this region is a flexible loop essential for the transfer of LPS from CD14 to monomeric MD-2 and for TLR4 activation at the cell surface [23].

A more detailed analysis revealed features that validate the potential quaternary structural rearrangement at acidic pH. The dimerisation surface of the TLR4-MD-2-LPS crystal structure appears to contain a high number of histidine residues. Given that the imidazole ring of histidine has a p*K*_a_ of 6.1, relatively small shifts in pH can change the average charge of this residue at physiologically relevant pH values. Hence, for pH values <6.0, the imidazole side-chain is mostly protonated and carries two N-H bonds with a positive charge. I propose that, in the acidic endosomes, the introduction of these positive charges leads to repulsion of the TLR4 chains and triggers an alternative dimerisation mode, with only one histidine (His587) potentially involved in formation of a salt bridge with Asp614.

**Figure 3:**
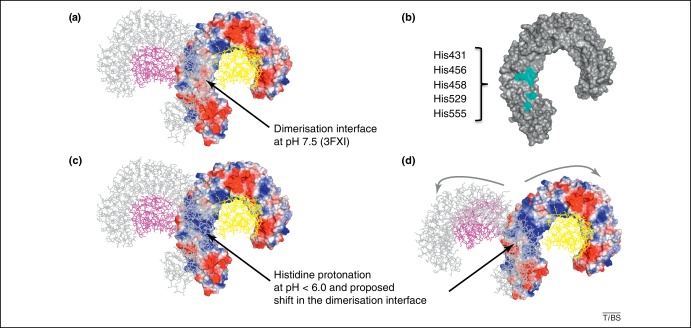
TLR4 contains histidine residues that modify its charge distribution upon endosomal acidification. **(a)** Electrostatic surface potential in the receptor chain of the TLR4-MD-2-LPS crystal structure (PDB code 3FXI) at the dimeric interface. Electropositive surface potentials are represented in blue and electronegative ones in red. **(b)** The dimerisation site of TLR4 contains five histidine residues that are sensitive to pH changes. **(c)** Below pH 6.0, histidine protonation may modify the electrostatic surface potential at the dimer interface. **(d)** The complex might undergo conformational changes upon endosomal acidification.

In the TLR4 crystal structure, three histidine residues (His431, His458 and His555) are involved directly in the interface, and two more are found in the immediate vicinity, His456 and His529. This represents a total of ten pH-sensitive residues within the dimerisation area (Figure 3). These histidines are strictly conserved across mammals, except for His456 and His458, which are specific to primates. His458 is located at the twofold symmetry axis of the TLR4 homodimer and forms π-stacking interactions with its counterpart. It has been suggested that nickel binding to TLR4 is mediated by His458 and His456 and leads to receptor activation in the absence of LPS [24]. Alanine scanning and transgenic expression of human TLR4 in TLR4-deficient mice have confirmed the importance of these residues in conferring nickel allergy. Importantly, most of us are not sensitive to nickel. In the light of my analysis, nickel sensitization could originate at the cell surface, because intracellular TLR4 would bear protonated histidines, unable to bind divalent metallic cations. It is extremely puzzling that MD-2 is required in nickel sensitization, because the key MD-2 residue Phe126 would be expected to prevent TLR4 dimerisation and subsequent activation [18,21,25,26]. Therefore, although nickel can trigger TLR4 signalling in recombinant systems, it is generally prevented *in vivo* by an unknown mechanism.

## Pathway-specific TLR4 ligands

A growing number of TLR4 ligands, such as allergenic nickel and the adjuvant monophosphoryl lipid A (MPLA), are pathway-specific. Whereas *Escherichia coli* LPS fully stimulates both pathways, nickel selectively activates the MyD88-dependent pathway and thus triggers inflammation and allergy [24]. I have discussed above a potential mechanism of nickel chelation of TLR4 at the cell surface, which would explain why nickel cannot elicit TRIF signaling at the acidic endosomes. By contrast, MPLA activates the TRIF pathway but is unable to trigger MyD88 signaling [27]. MPLA is an LPS derivative that lacks the phosphate in position 1 of the lipid-A headgroup and is 100-fold less toxic than LPS. Why is MPLA unable to activate the MyD88 pathway? Charge distribution is important for TLR4 activity [22], and the binding of LPS (which is negatively charged) creates an attractive force between TLR4 and MD-2. Therefore, I suggest that this force may be significantly weaker for MPLA compared to LPS at the cell surface, whereas the pH-driven conformational change that I hypothesize for TLR4 in the endosomes would allow MPLA to signal intracellularly as efficiently as LPS.

## CD14 is essential for intracellular TLR4 signaling

**Figure 4:**
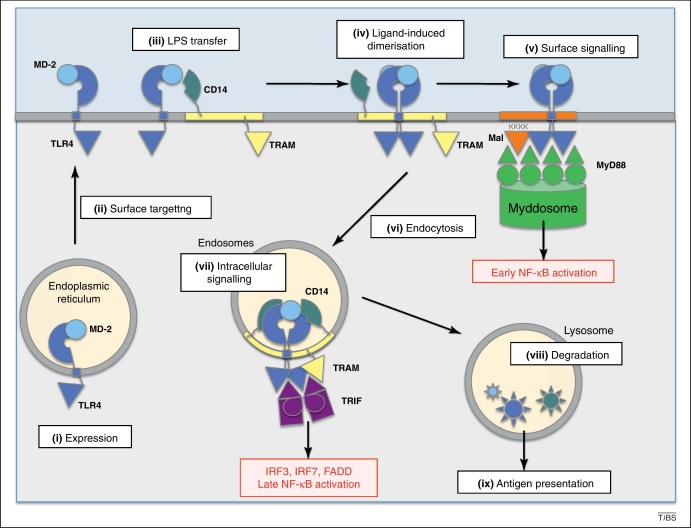
Proposed mechanism for TLR4 cellular targeting and signalling. **(i)** TLR4 is expressed in the endoplasmic reticulum. **(ii)** It relies on MD-2 among other protein partners for surface targeting. **(iii)** LPS is transferred from CD14 to TLR4-MD-2. **(iv)** Ligand binding triggers receptor dimerisation. **(v)** Mal has a phosphatidylinositol 4,5-bisphosphate (PIP2) binding motif (depicted as KKKK) that targets the receptor complex to a membrane microdomain. PIP2 is a minor phospholipid exclusively located at the plasma membrane. Surface signaling involves the myddosome and leads to early NF-κB activation. **(vi)** Ligand binding is also required for endocytosis in a dynamin-dependent mechanism. CD14 and TRAM have lipid-raft localization signals and are engulfed along the TLR4-MD-2-LPS complex. Mal is not translocated out of the membrane. I propose that, upon endosomal acidification, TLR4 undergoes a conformational change that brings its transmembrane domains closer together. In addition, the TIR domains might arrange slightly differently under the curved membrane of the endosomes, which would lead to a different stacking of the TIR domains that would allow TRAM recruitment. **(vii)** Endosomal signaling results in the recruitment of TRAM and TRIF in the case of LPS and MPLA (recruitment of TRAM but not TRIF for glycoprotein G from vesicular stomatitis virus VSV), and triggers a delayed NF-κB response. TRIF recruitment leads to the activation of IRF3, IRF7, NF-κB and FADD, respectively. **(viii)** In the lysosome, all endocytosed complexes are targeted for degradation and **(ix)** antigen presentation.

CD14 facilitates LPS transfer from the serum LPS-binding protein (LBP) to TLR4-MD-2 in a way that enhances, but is dispensable for, TLR4 surface signalling. However, CD14 serves a far more important intracellular role. *Heedless*, a mutation that causes CD14 to be produced as a C-terminally truncated protein, impairs activation of IRF3 in mice [28]. As the *Heedless* phenotype can be rescued by overexpression of soluble CD14, it has been suggested that CD14 enables a change or participates directly in the supramolecular structure of TLR4 that allows MyD88-independent signalling (Figure 4). Therefore, these results support the idea that TLR4 adopts different conformations at the cell surface and in the endosomes.

## From alternative receptor conformations to selective adaptor recruitment

Ligand binding leads to TLR4 dimerisation and the clustering of the signalling complexes in specific membrane microdomains (Figure 4). Whereas Mal targets the complexes to PIP2-rich areas, CD14 directs them to lipid rafts along with TRAM [13,15]. However, TRAM is not activated at the cell surface. I suggest that the lack of TRAM signalling at the cell surface can be explained by alternative conformations of the TLR4 dimers. As described above, at the acidic pH of the endosomes, the ectodomains might be tilted and their C-termini could be closer together. Given that the transmembrane region of TLR4 is separated from the ectodomain by only four residues [29,30], the conformational change might produce a shift in the transmembrane helix which, together with the increased membrane curvature of the endosomes (compared to that of the plasma membrane at the cell surface), would possibly alter the association of the cytosolic TIR domains (Figure 4). The different association of the TIR domains could be the reason why TRAM is recruited to the receptor complex in the endosomes and not at the cell surface.

## Concluding remarks

This Opinion proposes that the TLR4 dimer might undergo conformational changes at different cellular locations due to environmental factors such as pH, the presence of cell-specific regulators on either sides of the membrane and the segregation of the signalling components into membrane microdomains. Different dimerisation modes might in turn lead to the recruitment of different sets of adaptors, which determines the signalling output. Further work is clearly needed to test this hypothesis. The effect of pH can be assessed by crystallographic studies in acidic conditions, or by mutagenesis in which the histidine residues are changed into positively charged lysines or arginines to mimic the effect of endosomal acidification. The intracellular role of CD14 needs to be clarified along with that of the plethora of TLR4 regulators. Recently, the crystal structure of the complex formed between MD-1 and RP105 (a TLR-related molecule) has revealed a different dimerisation mode of the closest structural homologue of the TLR4-MD-2 complex [32,33]. More is to be expected with respect to TLR4 structure and function with the characterization of non-canonical ligands. Of particular interest is glycoprotein G from vesicular stomatitis virus (VSV) that has been shown to trigger a third pathway in endosomes based on the recruitment of TRAM in the absence of TRIF [31]. In keeping with this Opinion, the unusual signalling properties of VSV might be caused by a conformation distinct from that induced by LPS and MPLA.

TLR4 is a major pharmaceutical target in septic shock, inflammatory and autoimmune diseases, allergy and cancer. Anticipating different TLR4 dimerisation modes might be useful in rational drug design, because this technique is limited by the difficulty of predicting major conformational changes in proteins.

## Glossary

Adjuvant: A molecule that is added to a vaccine to amplify the immune response against the antigen. Mineral oil (Freund's adjuvant), aluminium hydroxide and, more recently, monophosphoryl lipid A (MPLA) have been used as adjuvants. Only MPLA functions via TLR4.
Cysteine-rich capping region: The conserved leucine residues that form the hydrophobic core of the first and the last leucine-rich repeat (LRR) are shielded from solvent exposure by cysteine-rich capping regions at the N- and the C-terminus of the LRR domain. They are referred to as LRRNT and LRRCT, respectively. In TLRs, the LRRNT is a β-hairpin stabilized by a single disulfide bond, the LRRCT adopts an α-β fold containing two disulfides.
Juxtamembrane region: The extracytoplasmic region located upstream and in proximity of the transmembrane region. In TLRs, the juxtamembrane region refers to the C-terminal cysteine-rich capping region (LRRCT).
Leucine-rich repeat (LRR): A short sequence of about 24 residues in TLRs in which leucine residues occupy conserved positions that are buried to form the hydrophobic core of the protein. Each repeat adopts a short β-strand and a loop conformation that might contain elements of secondary structure. LRR sequences are repeated throughout the extracytoplasmic domain of TLRs and form a solenoid tertiary structure (i.e. a coil) with a parallel β-sheet on the concave side and variable structural features on the lateral and the convex sides, which influence the degree of curvature of the protein. LRR motifs contain surface exposed residues that are highly variable, which makes it a versatile motif for recognition of vast array of ligands and diverse protein-protein contacts.
Type I membrane receptors: The type refers to the position of the N-terminus of the protein. Type I membrane receptors such as TLRs have an extracytoplasmic N-terminus and are anchored to the lipid membrane by a single-pass transmembrane α-helix.
