# Supplementary material for: Different dimerisation mode for TLR4 upon endosomal acidification?
Source: Trends Biochem Sci. 2012 Mar;37(3):92–8. doi: 10.1016/j.tibs.2011.11.003 (PMC3323831; doi:10.1016/j.tibs.2011.11.003)
Supplement: Supplementary file 1 [file mmc1.pdf]

Supplementary Material

A different dimerisation mode for TLR4 upon endosomal acidification?

Monique Gangloff
Department of Biochemistry, University of Cambridge, 80 Tennis Court Road, Cambridge CB2 1 GA, United Kingdom
Corresponding author: Gangloff, M. (mg308@cam.ac.uk)

Supplementary Figure 1. The 'tilted' TLR4 dimer is less compact than the crystal structure.
The complex is shown in sphere representation. (a) Side view, (b) a rotated side view, (c) top view. The T84-M85 loop of MD-2 is shown in orange and the TLR4 residues Arg460, Ala462, Gln484, Glu485 at the dimerization interface are shown in dark blue. Hydrophobic TLR4 residues F440 and F463 (ref. [1,2]) shown in red are located in close proximity to the exit of the MD-2 binding pocket.

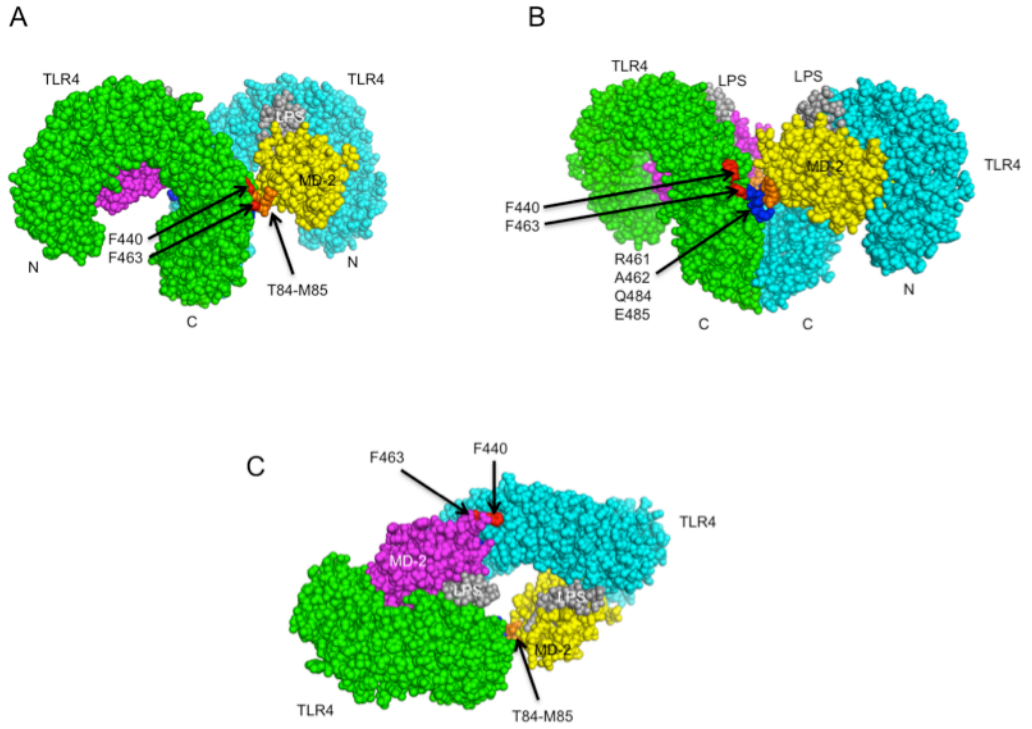

References:
1. Walsh, C., et al. (2008) Elucidation of the MD-2/TLR4 interface required for signaling by lipid IVa. *J Immunol* 181, 1245-1254.
2. Resman, N., et al. (2009) Essential roles of hydrophobic residues in both MD-2 and toll-like receptor 4 in activation by endotoxin. *J Biol Chem* 284, 15052-15060.

Supplementary Figure 2. The T84-M85 loop of MD-2 at the dimerisation interface undergoes a conformational change induced by LPS binding.
The LPS-induced conformational change of the T84-M85 loop, as well as the F126 loop, becomes apparent when the MD-2 molecules of the Eritoran antagonist complex (PDB accession code 2Z65, Eritoran shown in yellow) and the LPS agonist complex (PDB accession code 3FXI, LPS shown in gray) of both crystal structures are superimposed. The primary MD-2 binding site is conserved in both complexes, whereas dimerization is impaired in the Eritoran complex due to steric clashes between the TLR4 molecule at the dimer interface and the flexible loops of MD-2.

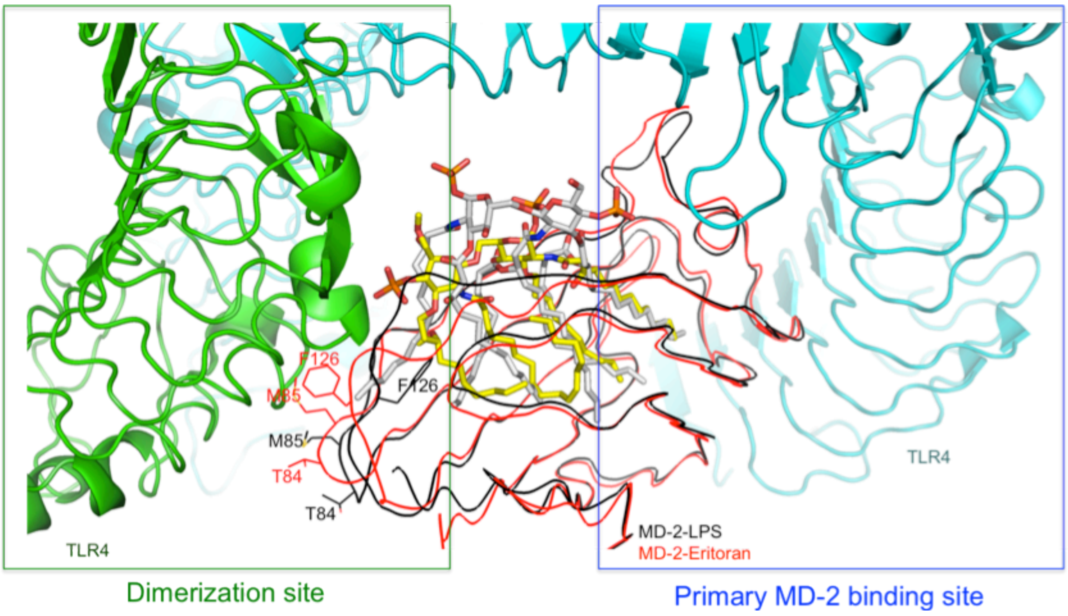

Supplementary Table 1. Storage and crystallization conditions of TLR complexes.
The fact that the Eritoran complex has been crystallized at acidic pH proves that the primary MD-2 binding site is unaffected by pH.

**TLR complexes**	**Storage buffers**	**Crystallization conditions**
hTLR4-hMD-2-LPS (PDB code 3FXI)	20mM Tris-HCl pH 8.0, 200mM NaCl	50mM MgCl2, 0.1M Na HEPES pH 7.5, 30% PEGmme 550
mTLR3-dsRNA (PDB code 3CYI)	20mM PIPES pH 5.5, 150mM NaCl	13% PEG3350, 5% Dextran 50000, 0.2M ammonium citrate pH 5.5
hTLR1-hTLR2-Pam$_3$CSK$_4$ (PDB code 2Z7X)	20 mM Tris HCl pH 8.0, 200 mM NaCl	0.2 M sodium citrate, 20% PEG3350
mTLR2-mTLR6-Pam$_2$CSK$_4$ (PDB code 3A79)	20 mM Tris HCl pH 8.0, 200 mM NaCl	2.0 M ammonium sulfate and 0.1 M MES pH 5.5
hTLR4-hMD-2-Eritoran (PDB code 2Z65)	?	0.2M M ammonium sulfate, 0.1 M Sodium Acetate pH 4.5, 20% PEG4000, 5% Ethanol
